# A novel system for gene silencing using siRNAs in rice leaf and stem-derived protoplasts

**DOI:** 10.1186/1746-4811-2-13

**Published:** 2006-06-29

**Authors:** Rebecca Bart, Mawsheng Chern, Chang-Jin Park, Laura Bartley, Pamela C Ronald

**Affiliations:** 1 Department of Plant Pathology, University of California at Davis, Davis, California, USA

## Abstract

**Background:**

Transient assays using protoplasts are ideal for processing large quantities of genetic data coming out of hi-throughput assays. Previously, protoplasts have routinely been prepared from dicot tissue or cell suspension cultures and yet a good system for rice protoplast isolation and manipulation is lacking.

**Results:**

We have established a rice seedling protoplast system designed for the rapid characterization of large numbers of genes. We report optimized methods for protoplast isolation from 7–14 day old etiolated rice seedlings. We show that the reporter genes luciferase GL2 and GUS are maximally expressed approximately 20 h after polyethylene glycol (PEG)-mediated transformation into protoplasts. In addition we found that transformation efficiency varied significantly with plasmid size. Five micrograms of a 4.5 kb plasmid resulted in 60–70% transformation efficiency. In contrast, using 50 μg of a 12 kb plasmid we obtained a maximum of 25–30% efficiency. We also show that short interfering RNAs (siRNAs) can be used to silence exogenous genes quickly and efficiently. An siRNA targeting luciferase resulted in a significant level of silencing after only 3 hours and up to an 83% decrease in expression. We have also isolated protoplasts from cells prepared from fully green tissue. These green tissue-derived protoplasts can be transformed to express high levels of luciferase activity and should be useful for assaying light sensitive cellular processes.

**Conclusion:**

We report a system for isolation, transformation and gene silencing of etiolated rice leaf and stem-derived protoplasts. Additionally, we have extended the technology to protoplasts isolated from fully green tissue. The protoplast system will bridge the gap between hi-throughput assays and functional biology as it can be used to quickly study large number of genes for which the function is unknown.

## Background

Genomics tools such as DNA sequencing, microarrays and yeast-two-hybrid assays have propelled the field of genetics forward at a remarkable rate, yet mechanisms for defining gene function lag behind. To date, even for model systems such as rice and Arabidopsis, only a fraction of the total genes have been studied in depth using classical genetics and molecular biology techniques [1]. Two common methods for gene characterization are 1) mutant screens, where an illustrative phenotype is sought to elucidate gene function, and 2) the insertion of a transgene into the plant chromosome through plant transformation. Although invaluable, these methods are labor intensive and thus, not suited for hi-throughput assays. The use of transient assays offers an opportunity to study large numbers of genes quickly. However, most transient assays have only been optimized for dicots. In this report we have developed a transient assay using rice, a model monocot, to isolate and manipulate leaf and stem-derived protoplasts.

C. E. Cocking first reported the isolation of protoplasts from a variety of plants and tissue types in 1965 [2,3]. Since then, the use of protoplasts has been shown to be an invaluable tool for many types of assays [4-12]. An elegant series of papers by Hattori et al. investigated phosphorylation and protein localization of the ABA response factor, TRAB1, using protoplasts prepared from rice suspension cell cultures [6-8]. Although suspension cell-derived protoplasts are appropriate for some experiments, they represent cells in an undifferentiated state and are therefore not suitable for cell biological questions. To address this drawback, many groups have begun preparing protoplasts from plant leaf and stem tissue including Arabidopsis, tobacco and maize [13]. Asai *et al*. used Arabidopsis mesophyll protoplasts to characterize the function of plant kinases and transcription factors acting downstream of FLS2, an Arabidopsis pathogen recognition receptor. Although various reports using dicot leaf and stem-derived protoplasts exist, this technology has been very limited in its extension to monocots and completely lacking for rice.

Here we combine the use of leaf and stem-derived rice protoplasts with short-interfering RNA (siRNA) technology in transient assays. The use of siRNAs is one of many new technologies stemming from the discovery of RNA interference (RNAi). First described in *C. elegans *by Tabara et al., RNAi is a mechanism used by eukaryotes to silence RNA transcripts [14]. Molecular biologists have exploited this endogenous process to silence genes of their choice using RNAi constructs and more recently, synthesized siRNAs. siRNAs are short (~21nt), double stranded regions of RNA that are incorporated into a silencing complex within a plant cell and direct the sequence specific cleavage of homologous mRNAs. To our knowledge only one report has shown the power of this technology in plant cells. In that study, Vanitharani, et al. transformed 3-day-old tobacco suspension cell-derived protoplasts with siRNAs targeting either Green Fluorescent Protein (GFP) or red fluorescent protein from Discosoma (DsRed2) and plasmids expressing both reporter genes (GFP and DsRed2). Fluorescence was measured and siRNA-mediated silencing resulted in a decrease in expression of 58% and 47%, respectively [15]. To date, siRNAs have not been used to silence genes in monocot and/or differentiated protoplasts.

Here we report the efficient isolation and transformation of rice leaf and stem protoplasts. We demonstrate efficient siRNA-mediated silencing of the firefly luciferase reporter gene and report a time course and concentration gradient for silencing. Our transformation efficiencies reach 60–70% while our silencing efficiency reached 83%. These efficiencies exceed those previously published for rice protoplasts and siRNA-mediated silencing, respectively [15]. Finally, because many cellular processes are light sensitive including disease resistance [16], we have extended protoplast isolation to fully green tissue. Although from Arabidopsis, protoplasts can be readily isolated from young, green plants, the limited literature that exists for monocots suggests that the use of etiolated or greening tissue is preferred (growing the plants in the dark and then moving them to light a short time before protoplast isolation) [13]. We have now shown that siRNA-mediated silencing can be applied to both etiolated tissue-derived protoplasts as well as protoplasts prepared from fully green tissue.

## Results

### Isolation of rice leaf and stem protoplasts

Protoplasts were isolated from three varieties of japonica rice: Kitaake, Taipei 309, and a transgenic Taipei 309 line carrying Xa21 [17]. All rice varieties tested yielded protoplasts at a similar rate (data not shown). Protoplast isolation was done as described [13] with changes noted in Methods. Twenty, two-week-old plants, digested in enzyme solution for 4 h, resulted in 1–5 × 10^6 ^cells. Cell quantification was done using a hemocytometer. After digestion, protoplasts were released with nearly 100% viability. The viability of the protoplasts was determined by staining with Evan's blue dye (stains dead cells blue) and observation under a light microscope (data not shown). Healthy protoplasts are round where as stressed or dying cells often appear irregular or lumpy.

### Transformation of rice leaf and stem protoplasts

**Figure 1:**
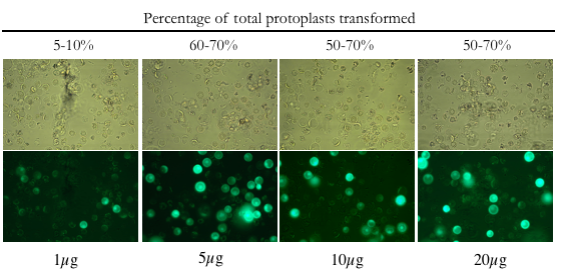
**Rice leaf protoplasts transformed with varying concentrations of a plasmid containing GFP**. Protoplasts were prepared from 2-week-old kitaake plants and transformed as described (Methods) with p35S-GFP. Images were taken at 40× magnification under either bright field (top) or a GFP specific filter (bottom). Pictures are representative of two independent experiments for each concentration of plasmid DNA.

To optimize transformation efficiency for our system, we experimented with different transformation conditions. We found that 1–5 × 10^6 ^cells/ml gave the highest level of luciferase expression. We also tested different transformation times (incubating protoplasts, DNA and PEG together for 5, 15 and 30 minutes) and found that 15 minutes was the optimum time for transformation (data not shown). To determine the optimum concentration of DNA for PEG-mediated transformation, plasmid p35S-GFP (Table 1) was transformed into Kitaake protoplasts at concentrations of 1, 5, 10, and 20 μg/10^6 ^cells/ml. Transformation efficiencies were calculated as described in Methods. There was no significant difference in protoplast survival, for the different concentrations of plasmid (Fig. 1). Using 1 μg of p35S-GFP plasmid DNA, transformation efficiency ranged from 5–10%. Using 5 μg of plasmid DNA, efficiency increased to 60–70%. Concentrations above 5 μg (10 or 20 μg) did not significantly improve transformation efficiency.

**Table 1:** Plasmid constructs and siRNAs summary. A list of plasmid and siRNA size, description and origin.

**Name**	**Nucleic acid type**	**Size (bp)**	**Description**	**Origin**
pLUC	Plasmid	~5500	Cauliflower mosaic virus (CaMV)35S promoter-luciferase GL2	[23]
p35S-GFP	Plasmid	~4500	35S (CaMV)-EGFP	[24]
pUbi-GFP	Plasmid	~12000	smGFP in Ubiquitin (Ubi)-pCambia 1300 backbone	This study
pGUS	Plasmid	~4500	Ubi-GUS	[22]
siLUC	siRNA	21	siRNA targeting luciferase GL2	Qiagen, USA

To test the effect of plasmid size on transformation efficiency, we compared pUbi-GFP (12 kb) with p35S-GFP (4.5 kb). With the larger plasmid, 1, 5 and 10 μg each gave 1–10% transformation efficiency where as either 25 μg or 50 μg gave about 25–30% transformation efficiency. These results indicate that smaller plasmids have higher transformation efficiency.

### Time course for reporter gene expression in rice leaf and stem protoplasts

**Figure 2:**
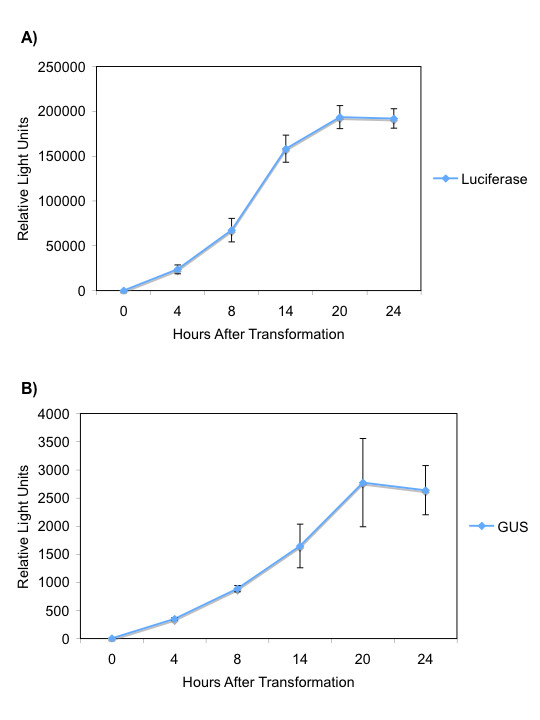
**Luciferase and GUS expression over time**. Protoplasts were prepared from 2-week-old kitaake plants and transformed as described (Methods). Luciferase (Top) or GUS (Bottom) activity was measured at 0, 4, 8, 14, 20, and 24 h post transformation. At each time point, cells were lysed by vortexing for one minute in lysis buffer (Methods) and frozen at -20°C until the experiment was complete. After the final time point, reporter gene activity was measured for all samples as described in Methods. Error bars represent 3 replicates at every time point.

To determine the amount of delay of gene expression after transformation, luciferase and GUS activity were assayed over time. Five μg of pLUC or pGUS was transformed into protoplasts and reporter gene activity was assayed at 0, 4, 8, 14, 20, and 24 h post transformation (Fig. 2). Significant expression of both reporter genes was seen by 4 h post transformation with peak activity at approximately 20 h. A decrease in luciferase activity was seen at 42 h post transformation (data not shown), possibly due to decreased protoplast viability. Three replicates were averaged and standard deviation from the mean was calculated. Two independent experiments showed similar results (Fig. 2)

### siRNA silencing in protoplasts

**Figure 3:**
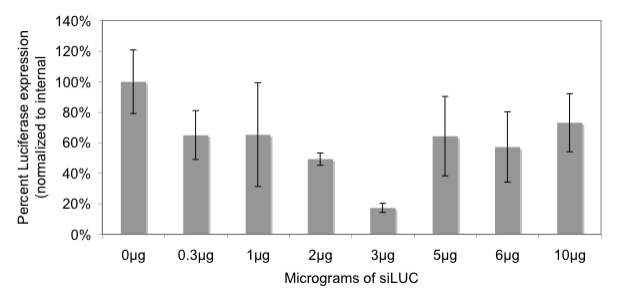
**Silencing of the luciferase gene by siRNAs at different concentrations**. Protoplasts were prepared from 2-week-old kitaake plants and transformed as described (Methods). Varying concentrations of siRNA to luciferase (0 μg, 0.3 μg, 1 μg, 2 μg, 3 μg, 5 μg, 6 μg and 10 μg) were transformed into protoplasts along with pLUC (10 μg) and pGUS (10 μg) as an internal control. Relative luciferase activity values for each concentration of siRNA were normalized to the observed GUS expression from the same set of protoplasts. Two independent experiments (separate preparations of protoplasts) were performed with similar outcomes. Each experiment contained two replicates for each concentration of siRNAs.

In a previous study, siRNA-mediated silencing was examined using tobacco suspension culture cells. Transformation was done with either 0.5 μg or 3 μg siRNAs and the latter proved more efficient [15]. To determine the optimum concentration of siRNAs needed to silence luciferase expression in rice seedling protoplasts, a range of concentrations (0, 0.3, 1, 2, 3, 5, 6 and 10 μg) of siLUC were co-transformed into protoplasts with 5 μg of pLUC. Additionally, 5 μg of pGUS was transformed into the protoplasts and served as an internal control for all our experiments. Each replicate was normalized by dividing luciferase activity by the corresponding GUS activity. Normalized values were used in subsequent statistical analyses. Figure 3 shows the effect of varying amounts of siRNAs targeting luciferase on total luciferase activity. The optimum amount of siLUC was 3 μg, which resulted in 17% remaining luciferase expression 16 h post transformation. Silencing with 5, or 6 μg siRNAs gave variable results but was consistently less efficient than 3 μg. At 10 μg, very little expression of both reporter genes was observed. We suspected that a lack of reporter gene expression correlated to decreased protoplast viability and that this was due to the lethal effect of siRNAs at high concentrations. This hypothesis was confirmed using Evan's blue dye as a vital stain (data not shown). Two independent experiments (different preparations of protoplasts) were done with similar outcomes. Each experiment contained 3 replicates for each concentration of siRNA.

**Figure 4:**
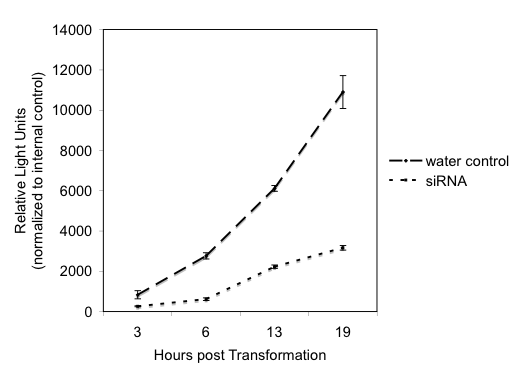
**siRNA silencing over time**. Protoplasts were prepared from 2-week-old kitaake plants and transformed as described (Methods). Top line: 5 μg of pLUC transformed into protoplasts as described. Bottom line: 5 μg pLUC and 3 μg of siRNA to luciferase co-transformed into protoplasts. Luciferase activity was measured after 3,6, 13 and 19 h. Each time point represents 3 replicates and standard errors are displayed.

We next determined the time scale on which genes can be silenced. Figure 2 shows that by 4 h post transformation, luciferase activity can be detected from pLUC and that expression peaks at 20 h. We therefore co-transformed pLUC and siLUC (as well as pGUS as an internal control) and assayed luciferase activity from 3–20 h. pLUC alone was included as a positive control (Fig. 4). Significant silencing was observed after only 3 h and continued to 19 h post transformation.

### Preparing protoplasts from green tissue

**Figure 5:**
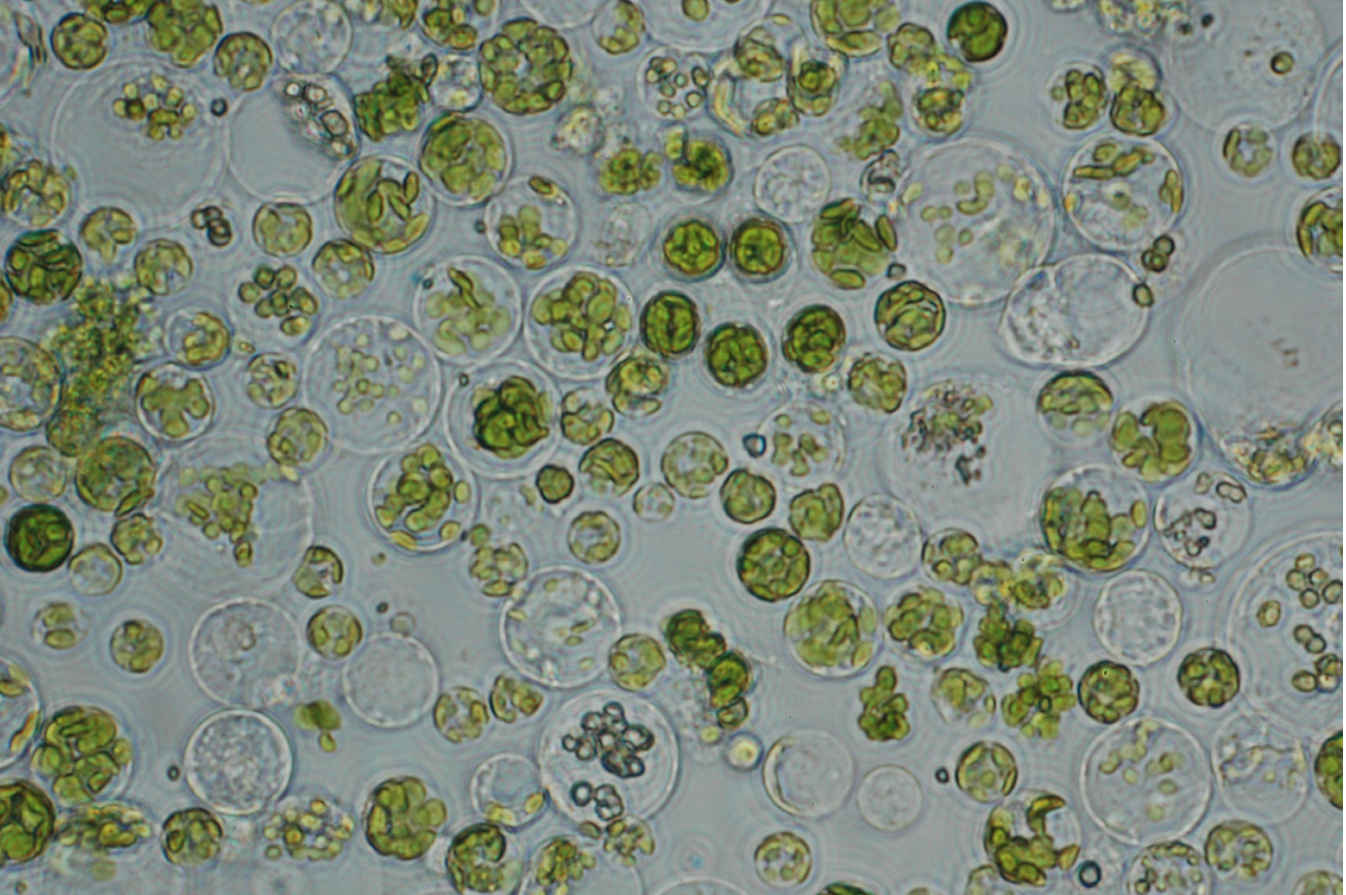
**Isolation of protoplasts from green tissue**. Protoplasts were prepared from 2-week-old kitaake plants and transformed as described (Methods). Picture was taken under bright field light using a Zeiss Axiovert 25 microscope with a 40× objective. Larger clear cells are derived from stem tissue whereas smaller chloroplast-filled cells were derived from green leaf tissue.

Many cellular processes are light sensitive and thus, we were interested in developing a method of isolating and manipulating protoplasts from green tissue. Using our protocol, we were able to efficiently isolate protoplasts from two-week old kitaake plants grown in full light conditions (16 h light, 8 h dark) (Fig. 5). Approximately 20 plants were harvested and treated with a cell wall removing enzyme solution as described in Methods. Twenty plants digested for 4 h in enzyme solution yielded 1–5 × 10^6 ^cells.

**Figure 6:**
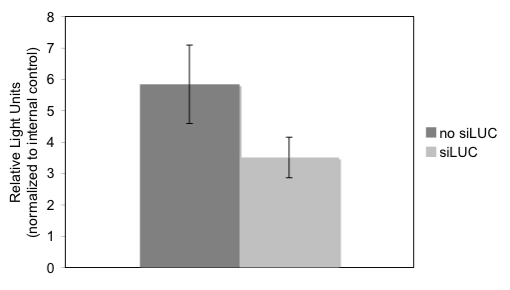
**Transformation and silencing in protoplasts prepared from green tissue**. Protoplasts were prepared as described (Methods). Protoplasts were suspended in Mmg solution at a concentration of 5 × 10^6 ^cells/ml. For transformation, 5 μg pLUC and 5 μg pGUS (as an internal control) were combined with 100 μl (5 × 10^6 ^cells/ml) of protoplasts and with or without 3 μg siLUC. Luciferase activity was measured as described 16 h after transformation.

Protoplasts prepared from fully green tissue were used in transformation experiments with pLUC and pGUS (as an internal control) as described in methods using the PEG-mediated transformation method. At the same time, silencing experiments were performed. Three micrograms of siLUC was transformed into protoplasts along with pLUC and pGUS. A 40% decrease in expression of pLUC was observed, indicating efficient silencing (Fig. 6). The silencing experiment was repeated three times with similar results.

Protoplasts prepared from etiolated or fully green tissue gave two easily distinguishable cell sizes. This difference was more obvious in protoplasts from green tissue as the smaller cells were generally filled with chloroplasts where as the larger cells were clear, containing a large central vacuole (Fig. 5). We hypothesized that the two different cells sizes represented a difference between leaf and stem cells. To test this hypothesis, two-week-old fully green tissue was separated into dark green leaf tissue and light green stem tissue. Protoplasts were prepared as described in Methods and the resulting cells were observed under the light microscope. As expected, the protoplasts derived from the stem tissue was enriched with larger clear cells where as the protoplasts from the green leaf tissue was enriched in smaller dark green cells (data not shown).

## Discussion

As genomics approaches yield large numbers of candidate genes, it becomes increasingly important to develop transient assays capable of evaluating gene function efficiently. The use of protoplasts has gained popularity in the last couple of years because of the ability to transiently express genes of interest [1,4-8,13,18-20]. This technique is especially important for plant species, such as rice, where VIGS (virus induced gene silencing) is not yet optimized [21]. Most of these studies have used protoplasts derived from cell suspension cultures or dicot tissue; there have been no reports of protoplast preparation from rice leaf and stem tissue. Here we report the development of a transient assay combining the use of rice leaf and stem-derived protoplasts with siRNA technology. This system is designed to bridge the gap between hi-throughput assays and functional biology and thus, one drawback is that protein behavior in intact plants may differ from protoplasts. Subsequent *in planta *experiments are needed to confirm results from transient assays.

We chose to utilize protoplasts prepared from growing plant tissue (leaf and stems) because these cells most closely represent the differentiated state appropriate for examining cellular processes. Some sources have suggested that electroporation is more efficient for monocots [13] however, in our hands, electroporation was limited to 50% transformation efficiency (data not shown) whereas using PEG-mediated transformation we achieved as high as 70% efficiency. We found that for rice leaf and stem protoplasts, 5 μg of p35S-GFP (4.5 kb) was sufficient to achieve high transformation efficiency (60–70%). However, with larger plasmids (12 kb), 50 μg of DNA yielded only 25–30% transformation efficiency. Along with plasmid size, a recent paper by De Sutter *et al*. suggests that optimum concentration of DNA may vary among cell types. The authors compared Arabidopsis and tobacco BY2 suspension cell cultures and report optimum amounts of plasmid DNA as 10 μg and 2 μg, respectively [1].

In the current study we report the ability to silence genes in protoplasts using siRNA technology. Using a siRNA targeting luciferase, we obtained up to 83% silencing as compared to the non-silenced control. To our knowledge there is only one other report using siRNAs to silencing genes in protoplasts. That study used tobacco suspension cells and obtained 58% and 47% silencing using siRNAs targeting GFP and DsRed2, respectively [15]. A similar study by An et al. (2003) used longer (~100 bp) pieces of dsRNA to silence exogenous genes. The authors achieved between 81% and 92% silencing using Arabidopsis suspension or mesophyll cells, respectively [18]. Another study used barley aleurone cell protoplasts and inverted repeat RNAi constructs to transiently silence the GUS reporter gene. The authors report approximately 88% silencing [20]. Taken together these reports suggest that protoplasts prepared from differentiated cells allow for higher levels of silencing. Additionally, silencing efficiency achieved using 100 bp of dsRNA or siRNAs is comparable. However, because siRNAs target a small region they may prove more gene specific than longer dsRNA since a shorter sequence should correlate with less chance of overlapping a related gene.

With the aim of developing a protoplast system to study diverse signaling pathways, many of which are light sensitive, we have established a protocol for preparing protoplasts from green tissue. High levels of transformation were readily achievable as well as efficient silencing. Interestingly, two main cells sizes resulted from our protoplast preparations. We determined that the smaller chloroplast-filled cells were derived from the green leaf tissue and the larger clear cells were from the stem. However, both tissues gave some cells of each size. We note that the inside of the stem of a growing rice plant contains younger emerging leaves. Likewise, a rice leaf contains a central midrib whose cells may more closely resemble the stem than the leaf. This may explain the overlap in cell size.

One of the potential uses of this assay is deducing signaling cascades by silencing candidate genes and assaying for downstream gene expression. Interestingly, in the last 2 years the price of synthesized siRNAs has been cut in half and the list of validated siRNAs targeting specific genes is growing rapidly especially in the animal systems. Additionally, the demonstrated ability of siRNAs to silence genes in a concentration dependent manner opens up new opportunities to study gene function, for example, by studying genes that when completely silenced would result in the death of the cell.

## Conclusion

The use of siRNAs and transient assays hold great promise for increasing the speed at which genes can be studied, bridging the gap between the large data sets coming from hi-throughput assays and the time consuming and laborious but absolutely essential outcomes of *in planta *investigations. Here we report the first protocol for efficient isolation, transformation and silencing of rice leaf and stem protoplasts from both etiolated and fully green tissue. We have characterized aspects of this system including optimum amounts of DNA for transformation and RNA for silencing as well as time courses for gene expression and silencing. This system will be of use for investigating rice gene function in response to a variety of abiotic and biotic conditions.

## Methods

### Plant material

Plants were grown in plastic pots filled with soil either in the light or in the dark at 28°C. Rice (Oryzae sativa L. japonica) varieties were as follows: Taipei 309 (TP309), transgenic Taipei 309-I106(Xa21)[17], Kitaake.

### Plasmids and siRNAs

pLUC, pGUS and p35S-GFP have been described previously [22-24]. pUbi-GFP was made by cloning the GFP fragment (smGFP2) from p35S-GFP into Ubi-pCambial300 [16]. Plasmid DNA was purified using the Qiagen Maxi Plasmid kit (Qiagen, USA). Pre-designed siRNAs targeting luciferase GL2 (target sequence: AACGUACGCGGAAUACUUCGA) were purchased from Qiagen (USA).

### Protoplast preparation

Our protoplast isolation protocol was based on the protocol for maize protoplasts provided online by J. Sheen's laboratory  with several changes. Rice seeds were grown as stated above. Between 7 and 14 days post germination, plants were ~4–8 inches tall. Leaf and stem tissue was cut into 0.5 mm pieces using very sharp razors. Tissue was immediately incubated in enzyme solution (0.6 M mannitol, 10 mM MES (pH 5.7), 1.5% Cellulase RS, 0.75% Macerozyme, 0.1% BSA, 1 mM CaC12, 5 mM β-mercaptoethanol and 50 μg/ml carbenicillin) for 4 h in the dark under gentle shaking (40 rpm). After incubation, protoplasts were passed through a 35 μm nylon mesh filter. One volume of W5 solution (154 mM NaCl, 125 mM CaC12, 5 mM KC1, 2 mM MES (pH 5.7)) was added and the solution was centrifuged for 5 minutes at 1500 rpm to pellet the protoplasts. Cells were re-suspended in Mmg solution [13] (0.6 M mannitol, 15 mM MgC12, 4 mM MES (pH 5.7)) for PEG-mediated transformation at 10^6 ^cells/ml. Cells were quantified using a hemocytometer. For transformation, 40% PEG (0.6 M mannitol, 100 mM CaC12, 40% v/v PEG 3350) was added to the protoplasts for 15 minutes. Cells were washed 1× with 10 volumes of W5 and then re-suspended in incubation solution (0.6 M mannitol, 4 mM MES (pH 5.7), 4 mM KC1). Cells were incubated at 28°C in the dark overnight.

### Luciferase assay

Luciferase activity was quantified using the Luciferase Assay System available from Promega and the Auto Lumat LB 953 luminometer from EG&G Berthold. CCLR lysis buffer was added to the protoplasts before they were vortexed for 1 minute to lyse the cells. Cellular debris was spun down in a microcentrifuge and the supernatant was removed. 100 μl of luciferase substrate was automatically applied to 20 μl of supernatent. Luminescence was read for 10 seconds.

### GUS assay

GUS expression was quantified using the TBS-380 Mini-Fluorometer (Turner BioSystems). 5× lysis buffer was added to the protoplasts before they were vortexed for 1 minute to lyse the cells. Cellular debris was spun down in a microcentrifuge and the supernatant was removed. Supernatant was combined with 4 mM MUG in a 1:1 ratio and was incubated at 37°C for 1 h. The reaction was stopped by adding 1 ml 0.2 M Na2CO3. The fluorometer was calibrated using 10 μl 10 μM 4-MU.

### Microscopy

Transformation efficiency was calculated by counting the number of bright green (GFP expressing) protoplasts divided by the total number of cells in one representative microscope field. Five microscope fields were averaged to represent one experiment. Two independent experiments were performed with similar results and combined to give an average range of transformation efficiencies. All microscopy was performed using the Zeiss Axiovert 25 Fluorescent microscope. Images were taken using the attached Nikon D70s digital camera with the Nikon Capture 4 software. GFP fluorescence was visualized under the Zeiss GFP specific filter cube 38HE (excitation: BP 470/40; beamsplitter: FT 495; emission: BP525/50)

## Competing interests

The author(s) declare that they have no competing interests.

## Authors' contributions

RB optimized and characterized the protoplast system and drafted the manuscript. MS and CP created constructs, provided general direction and contributed to the manuscript. LE performed additional characterization experiments, and contributed to the manuscript. PCR initiated and guided the project and co-wrote the MS.

## Note added in proof

The establishment of a novel rice protoplast system for gene silencing is also presented in a paper by Chen *et al*. 2006. *Molecular Plant Pathology*. 7(5): In press.

## References

[B1] De Sutter V, Vanderhaeghen R, Tilleman S, Lammertyn F, Vanhoutte I, Karimi M, Inze D, Goossens A, Hilson P (2005). Exploration of jasmonate signalling via automated and standardized transient expression assays in tobacco cells. Plant J.

[B2] Gregory DW, Cocking EC (1965). The Large-Scale Isolation Of Protoplasts From Immature Tomato Fruit. J Cell Biol.

[B3] Cocking EC (1974). The isolation of plant protoplasts. Methods Enzymol.

[B4] Asai T, Stone JM, Heard JE, Kovtun Y, Yorgey P, Sheen J, Ausubel FM (2000). Fumonisin Bl-induced cell death in arabidopsis protoplasts requires jasmonate-, ethylene-, and salicylate-dependent signaling pathways. Plant Cell.

[B5] Asai T, Tena G, Plotnikova J, Willmann MR, Chiu WL, Gomez-Gomez L, Boller T, Ausubel FM, Sheen J (2002). MAP kinase signalling cascade in Arabidopsis innate immunity. Nature.

[B6] Hattori T, Terada T, Hamasuna S (1995). Regulation of the Osem gene by abscisic acid and the transcriptional activator VP1: analysis of cis-acting promoter elements required for regulation by abscisic acid and VP1. Plant J.

[B7] Hobo T, Kowyama Y, Hattori T (1999). A bZIP factor, TRAB1, interacts with VP1 and mediates abscisic acid-induced transcription. Proc Natl Acad Sci USA.

[B8] Kagaya Y, Hobo T, Murata M, Ban A, Hattori T (2002). Abscisic acid-induced transcription is mediated by phosphorylation of an abscisic acid response element binding factor, TRAB1. Plant Cell.

[B9] Sugimoto K, Takeda S, Hirochika H (2003). Transcriptional activation mediated by binding of a plant GATA-type zinc finger protein AGP1 to the AG-motif (AGATCCAA) of the wound-inducible Myb gene NtMyb2. Plant J.

[B10] Meyer A, Eskandari S, Grallath S, Rentsch D (2006). AtGATl, a high affinity transporter for gamma-aminobutyric acid in Arabidopsis thaliana. J Biol Chem.

[B11] Pih KT, Yi MJ, Liang YS, Shin BJ, Cho MJ, Hwang I, Son D (2000). Molecular cloning and targeting of a fibrillarin homolog from Arabidopsis. Plant Physiol.

[B12] Yao N, Greenberg JT (2005). Arabidopsis ACCELERATED CELL DEATH2 Modulates Programmed Cell Death. Plant Cell.

[B13] Sheen J (2001). Signal transduction in maize and Arabidopsis mesophyll protoplasts. Plant Physiol.

[B14] Tabara H, Grishok A, Mello CC (1998). RNAi in C. elegans: soaking in the genome sequence. Science.

[B15] Vanitharani R, Chellappan P, Fauquet CM (2003). Short interfering RNA-mediated interference of gene expression and viral DNA accumulation in cultured plant cells. Proc Natl Acad Sci USA.

[B16] Chern M, Fitzgerald HA, Canlas PE, Navarre DA, Ronald PC (2005). Overexpression of a rice NPR1 homolog leads to constitutive activation of defense response and hypersensitivity to light. Mol Plant Microbe Interact.

[B17] Song WY, Wang GL, Chen LL, Kim HS, Pi LY, Holsten T, Gardner J, Wang B, Zhai WX, Zhu LH (1995). A receptor kinase-like protein encoded by the rice disease resistance gene, Xa21. Science.

[B18] An CI, Sawada A, Fukusaki E, Kobayashi A (2003). A transient RNA interference assay system using Arabidopsis protoplasts. Biosci Biotechnol Biochem.

[B19] An CI, Sawada A, Kawaguchi Y, Fukusaki E, Kobayashi A (2005). Transient RNAi induction against endogenous genes in Arabidopsis protoplasts using in vitro-prepared double-stranded RNA. Biosci Biotechnol Biochem.

[B20] Zentella R, Yamauchi D, Ho TH (2002). Molecular dissection of the gibberellin/abscisic acid signaling pathways by transiently expressed RNA interference in barley aleurone cells. Plant Cell.

[B21] Holzberg S, Brosio P, Gross C, Pogue GP (2002). Barley stripe mosaic virus-induced gene silencing in a monocot plant. Plant J.

[B22] Christensen AH, Quail PH (1996). Ubiquitin promoter-based vectors for high-level expression of selectable and/or screenable marker genes in monocotyledonous plants. Transgenic Res.

[B23] Chern MS, Bobb AJ, Bustos MM (1996). The regulator of MAT2 (ROM2) protein binds to early maturation promoters and represses PvALF-activated transcription. Plant Cell.

[B24] Park CJ, Kim KJ, Shin R, Park JM, Shin YC, Paek KH (2004). Pathogenesis-related protein 10 isolated from hot pepper functions as a ribonuclease in an antiviral pathway. Plant J.

